# Regulation of Adult Mammalian Neural Stem Cells and Neurogenesis by Cell Extrinsic and Intrinsic Factors

**DOI:** 10.3390/cells10051145

**Published:** 2021-05-10

**Authors:** Shuzo Matsubara, Taito Matsuda, Kinichi Nakashima

**Affiliations:** Department of Stem Cell Biology and Medicine, Graduate School of Medical Sciences, Kyushu University, 3-1-1, Maidashi, Higashi-ku 812-8582, Fukuoka, Japan; mtsbr@scb.med.kyushu-u.ac.jp

**Keywords:** neurogenesis, neural stem cells, direct reprogramming, neurodegenerative diseases, injury, neurons, astrocytes, microglia

## Abstract

Tissue-specific stem cells give rise to new functional cells to maintain tissue homeostasis and restore damaged tissue after injury. To ensure proper brain functions in the adult brain, neural stem cells (NSCs) continuously generate newborn neurons that integrate into pre-existing neuronal networks. Proliferation, as well as neurogenesis of NSCs, are exquisitely controlled by extrinsic and intrinsic factors, and their underlying mechanisms have been extensively studied with the goal of enhancing the neurogenic capacity of NSCs for regenerative medicine. However, neurogenesis of endogenous NSCs alone is insufficient to completely repair brains damaged by neurodegenerative diseases and/or injury because neurogenic areas are limited and few neurons are produced in the adult brain. An innovative approach towards replacing damaged neurons is to induce conversion of non-neuronal cells residing in injured sites into neurons by a process referred to as direct reprogramming. This review describes extrinsic and intrinsic factors controlling NSCs and neurogenesis in the adult brain and discusses prospects for their applications. It also describes direct neuronal reprogramming technology holding promise for future clinical applications.

## 1. Introduction

**Figure 1:**
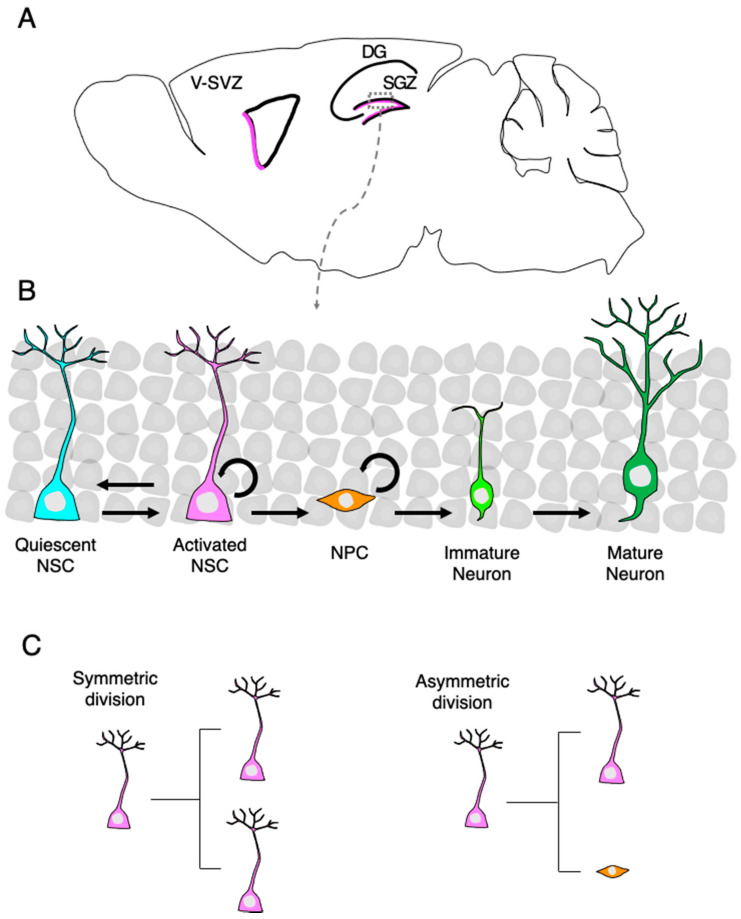
Neurogenesis of neural stem cells (NSCs) in the adult brain. (**A**) A sagittal section view of an adult rodent brain highlighting the two neurogenic regions, the subgranular zone (SGZ) in the dentate gyrus (DG) of the hippocampus and the ventricular-subventricular zone (V-SVZ) of the lateral ventricle. (**B**) Magnified view of the area outlined by the rectangle in (**A**). New granule neurons in the DG are generated through several consecutive developmental stages. Quiescent neural stem cells (NSCs) enter an active state in response to extrinsic stimuli and subsequently generate neural progenitor cells (NPCs). NPCs give rise to immature dentate granule neurons, which migrate into the granule cell layer and become functionally mature neurons. (**C**) NSCs undergo symmetric or asymmetric cell division; both daughter cells are the same NSCs in symmetric cell division, whereas one NSC and one NPC are produced in asymmetric division.

The subgranular zone (SGZ) of the dentate gyrus (DG) in the hippocampus and ventricular subventricular zone (V-SVZ) lining the lateral ventricle retain adult neural stem cells (NSCs) that can generate new neurons and glial cells (Figure 1A,B) [1]. Adult neurogenesis in humans, especially in the hippocampus, remains yet to be proven [2,3,4], whereas it is accepted to occur throughout life in rodents [5,6]. NSCs reside predominantly in a mitotically dormant, quiescent state and are activated in response to environmental inputs. Once NSCs are activated, they proliferate in two different modes of division: symmetric division generates two NSCs that return to quiescence for the maintenance of NSC pools, and asymmetric division generates one NSC and one neural progenitor cell (NPC) (Figure 1C). NPCs have a high proliferative capability that allows their expansion and also have the ability to differentiate into immature neurons. These immature neurons subsequently become mature dentate granule neurons and functionally integrate into pre-existing neuronal networks [7]. Adult neurogenesis in the hippocampus contributes to hippocampus-dependent cognitive functions and is involved in several neurological disorders, including age-related cognitive decline, major depressive disorders, and medial–temporal lobe epilepsy [8,9,10]. Therefore, elucidation of the mechanisms underlying life-long regulation of NSC’s behavior and neurogenesis is important for the conception and development of therapeutic strategies to overcome diseases caused by impaired adult neurogenesis.

Neuronal regeneration is a prerequisite for recovering brain dysfunction caused by neuronal damage or loss. Even in pathological conditions such as after ischemic injury, NSCs retain the potential to produce new neurons; however, their number is small due to low neurogenic efficiency and is insufficient to fully reverse brain impairments. Therefore, other strategies to efficiently replenish the lost neurons have been greatly desired.

Direct reprogramming is a technology to convert somatic cells from the original lineage to another by manipulating the expression of cell lineage-specific transcription factors that can rewrite epigenetic signatures, such as DNA methylation and histone modifications. Mouse fibroblasts, for example, have been converted to neurons in vitro by the forced expression of transcription factors Ascl1, Brn2, and Myt1l [11], all of which are known to regulate neurogenesis of NSCs. Moreover, recent advances in direct reprogramming have made it possible to induce in vivo neurogenesis from non-stem cells in the adult brain by single or combinatorial expression of transcription factors, enabling neuronal regeneration with low risk of immunogenicity and tumorigenesis [12]. This review outlines the mechanism underlying the regulation of neurogenesis from stem and non-stem cells in the adult brain and discusses future prospects of direct reprogramming technology to treat neurological disorders.

## 2. Neurogenesis from Adult NSCs

### 2.1. Extrinsic Signals Regulating NSC Behavior

Adult neurogenesis of NSCs in the DG occurs on the hilar side of the granule cell layer in a two- to three-cell-layer thick region designated as SGZ. The process of adult neurogenesis in the hippocampus starts with the Nestin-, Gfap- and SRY-box 2 (Sox2)-expressing quiescent NSCs, which are also referred to as radial glia-like cells because of their morphology and ontogeny. The decision of whether NSCs remain quiescent or enter an active state is controlled by niche cells via direct cell-cell contacts and secreted molecules. Notch is a representative factor regulating NSC behavior through cell-cell contacts. Notch ligand Jagged1 (Jag1) is expressed by NPCs and granule neurons in the DG [13]. Upon binding of this ligand, the cleaved and activated Notch intracellular domain is released from the cell membrane into the cytoplasm, translocates to the nucleus, and interacts there with the DNA-binding CSL protein (Rbpj in mice) to induce expression of target genes associated with NSC quiescence, such as hairy and enhancer of split (Hes) family genes [14]. Recent studies reported that *Notch2* is highly expressed in adult hippocampal NSCs and its loss activates quiescent NSCs in the DG. This aberrant NSC activation eventually leads to exhaustion of the NSC pool [15], which is similar to the phenotype of adult mice with conditional deletion of *Rbpj* in NSCs [16], suggesting that the Notch signaling pathway mediated by Notch2 participates in maintaining NSC quiescence.

The quiescence of tissue stem cells, including NSCs, is maintained by various common secreted molecules derived from local niche cells in each different tissue. Wnt, secreted from NSCs and astrocytes in the neurogenic niche, induces the activation of quiescent NSCs, similarly to other tissue quiescent stem cells [17,18]. Bone morphogenetic proteins (BMPs), on the other hand, are well-studied factors associated with NSC quiescence. BMPs are secreted from granule neurons and NSCs [19], inhibit NSC proliferation, and induce NSC quiescence to maintain the NSC pool for a long period [20]. In addition to BMPs, milk-fat globule-epidermal growth factor EGF factor 8 (Mfge8) has an important role in maintaining NSC quiescence. Mfge8 secreted by SGZ NSCs and astrocytes maintains NSC quiescence through integrinβ1-mediated mechanistic target of rapamycin (mTOR) signaling in autocrine and paracrine manners [21]. Sonic hedgehog (Ssh) is a pleiotropic signaling protein and is important for the proliferation and dorsoventral specification of cells during neural development [22]. Ssh is secreted from mossy fibers in the hilus of the adult hippocampus. The Ssh receptor, Patched, is expressed in adult NSCs [23]. Ablation of Shh in the adult DG resulted in increased proliferation of NSCs, suggesting that Ssh inhibits NSC activation in the adult DG [24]. By contrast, suppression of Shh signaling was reported to impair the expansion of long-lived NSCs due to the precocious transition of NSCs into a quiescent state during DG development, suggesting that Shh signaling promotes NSC activation and proliferation to expand the NSC pool in the early postnatal stage [25].

Neurotransmitters, such as serotonin, dopamine, glutamate, acetylcholine, noradrenaline, and gamma-aminobutyric acid (GABA), have been shown to regulate the quiescent to active state transition of NSCs as well as subsequent neurogenesis in the adult DG [26]. Recent advances in optogenetics have enabled researchers to further uncover neuronal circuitry mechanisms controlling NSC behavior spatiotemporally through these neurotransmitters. Using the combination of optogenetics and lineage tracing technology, recent studies have identified DG parvalbumin (PV)-positive interneurons acting as a cellular niche component that signals to quiescent NSCs through GABA type A receptors (GABA_A_Rs) in an activity-dependent fashion in the adult DG. Moreover, optogenetic control of DG PV-positive neuron activity, but not somatostatin- and vasoactive intestinal polypeptide-positive interneurons, dictates the NSC’s decision of whether they remain quiescent or enter activation states [27]. Furthermore, the dentate PV interneurons receive distal inputs from medial septal GABAergic neurons and are depolarized to secrete GABA, which promotes NSC quiescence [28]. Diazepam binding inhibitor (DBI), a factor that binds with high affinity to the GABA_A_R and dampens GABA activity, is known as a regulator of NSC activation. NSCs in the hippocampus express DBI, which negatively modulates GABA_A_ receptor signaling to promote NSC proliferation [29]. Cholecystokinin (CCK), a neuropeptide released from interneurons in the hilus is also known to enhance NSC proliferation through glutamatergic signaling mediated by local astrocytes in the DG. Released CCK stimulates CCK2 receptor-expressing astrocytes and promotes glutamate secretion, increasing NSC proliferation and neurogenesis. Conversely, reducing CCK release induces astrocyte reactivation, accompanied by the secretion of pro-inflammatory cytokines, and impairs NSC proliferation [30]. Taken together, these facts indicated that neurotransmitters also contribute to the regulation of NSC behavior in the adult DG.

The extracellular matrix (ECM) is a complex of secreted molecules, including glycoproteins and proteoglycan, that exists in the basement membrane lining tissues and in the intercellular spaces, and provides a functional scaffold for maintaining signaling gradients and stiffness. In the brain, the ECM plays critical roles in neuronal migration, axon outgrowth, myelination, synaptogenesis, synaptic plasticity, and NSC performance. Members of the transmembrane protein integrin family form a complex composed of an α and β subunit that allows cells to recognize and communicate with the ECM. The largest integrin subfamily is composed of complexes containing integrin β1 (Itgb1), which is highly expressed in NSCs in the adult DG. Specific deletion of *Itgb1* in adult NSCs resulted in extensive cellular disorganization of the SGZ as well as the non-neurogenic region in the DG. *Itgb1*-deficient NSCs rapidly lost their NSC properties and differentiated into astrocytes, indicating that Itgb1 maintains the NSC population and prevents the commitment of NSCs into the astrocytic lineage [31]. ECM signaling has also been reported to activate integrin-linked kinase (Ilk) via Itgb1 in adult NSCs. Conditional deletion of *Ilk* in the adult NSC increased NSC proliferation, although disorganization of the DG did not occur [32]. Stiffness, a major topographical index of tissues, is determined by the components of the ECM. A recent study showed that the niche stiffness affects the fate decision of NSCs via stretch-activated cation channel Piezo1 [33], which activates Yap, a mechanoreactive transcription coactivator known to induce NSC quiescence [34]. These results suggest that the interaction between the ECM and the proteins recognizing it on the cell surface provokes signaling cascades in NSCs to determine which direction, i.e., quiescence or activated state, they head to.

The above-described cell-extrinsic factors and their functions in the regulation of adult NSCs and neurogenesis are summarized in Table 1.

### 2.2. Cell Intrinsic Factors Regulating NSC Behavior

The multiple cell-extrinsic factors influence NSC behavior in collaboration with cell-intrinsic transcription factors [35]. *Hes* family genes, for instance, act as effectors of Notch signaling, as mentioned above, and induce NSC quiescence by suppressing the expression and function of *achaete-scute family bHLH transcription factor 1* (*Ascl1*), a well-known NSC activator. Hes1 expression oscillates and drives cyclic expression of Ascl1 in active NSCs, whereas sustained high expression of Hes1 in NSCs induces persistent suppression of Ascl1 expression, leading to quiescence of NSCs [36]. In support of this, conditional deletion of *Ascl1* in adult NSCs resulted in permanent quiescence of NSCs and loss of their responsiveness to activating stimuli such as glutamatergic signaling evoked by kainic acid [35]. The quiescent state of NSCs is also maintained by the inhibitor of DNA binding (Id) proteins whose genes are targets of BMP signaling. Id proteins can directly interact with Hes1 and inhibit the negative autoregulation of Hes1, contributing to sustained quiescence of NSCs [37]. Moreover, Id4, expressed in quiescent NSCs, sequesters Ascl1 heterodimerization partner E47 and promotes Ascl1 protein degradation [15,38,39]. Besides the regulation by Hes1 and Id4, the Ascl1 level is regulated at the protein level by other factors, such as HECT, UBA, and WWE domain containing 1 (Huwe1) [35,40]. Huwe1, an E3-ubiquitin ligase, induces polyubiquitination of Ascl1 and promotes its proteasomal degradation. Conditional deletion of *Huwe1* in adult NSCs extended the Ascl1 protein half-life, leading to the loss of their ability to return to the quiescent state and thus to constitutively activated adult NSCs [40].

The phosphoinositide-3-kinase (PI3K)-Akt-mTOR1 pathway in adult NSCs is also implicated in the regulation of their maintenance of quiescence and activation. Mfge8 binds to Itgb and activates phosphatase and tensin homolog (Pten), which is a major negative regulator of PI3K activation [41], resulting in the inhibition of Akt activation. Therefore, Mfge8 suppresses Akt-mediated activation of mTOR, which is required for NSC proliferation induced by PI3K-Akt activation [41]. In this context, it has been reported that the ablation of *Pten* induced NSC activation in the adult DG [42,43], suggesting that Pten maintains NSC quiescence. The mechanism of the PI3K-Akt-mTOR pathway-mediated regulation of proliferation in adult NSCs is not fully understood, but in fibroblasts, it is reported that Akt leads to GSK-3 phosphorylation, which allows for β-catenin and cyclin D1 activation to promote transcription and cell cycle progression [44]. Akt is also known to phosphorylate and inactivate forkhead box O3 (Foxo3), a key transcription factor for preserving quiescent NSCs in the adult DG [45,46,47]. Since Foxo3 shares target genes with Ascl1, it suppresses the expression of Ascl1 target genes associated with cell cycle progression. Therefore, Foxo3 induces and/or preserves the quiescent state of NSCs by inhibiting their Ascl1-mediated activation state entry [48]. Foxo3 has also been shown to upregulate quiescence-associated genes in NSCs, such as those involved in reactive oxygen species (ROS)-detoxification [45,46,49]. Foxo3-deficient NSCs have an increased intracellular ROS level due to the downregulation of ROS-detoxifying enzyme genes. The increased ROS level enhances NSC proliferation and neurogenesis, which are dependent on the PI3K-Akt axis [50].

It has been shown that when the transition from the quiescent to the activated state of adult NSCs occurs, they switch the metabolic system they use to obtain energy by using glucose from glycolytic to oxidative metabolism in the mitochondria [51,52,53,54]. Quiescent NSCs in the DG also gain energy produced by fatty acid oxidation [53]. Strikingly, inhibition of fatty acid oxidation is sufficient to pull NSCs out of quiescence, whereas inhibition of lipogenesis decreases the proliferation of NSCs [52]. In accord with these findings, a recent single-cell RNA-seq study revealed that the expression of genes associated with fatty acid metabolism and glycolysis is enriched in quiescent NSCs [55].

Table 2 summarizes the aforementioned functions of these cell-intrinsic factors regulating NSC behavior.

### 2.3. Live Imaging Elucidates Cell Division Patterns and Long-Term Self-Renewal Potential of the Adult NSCs in the DG

**Figure 2:**
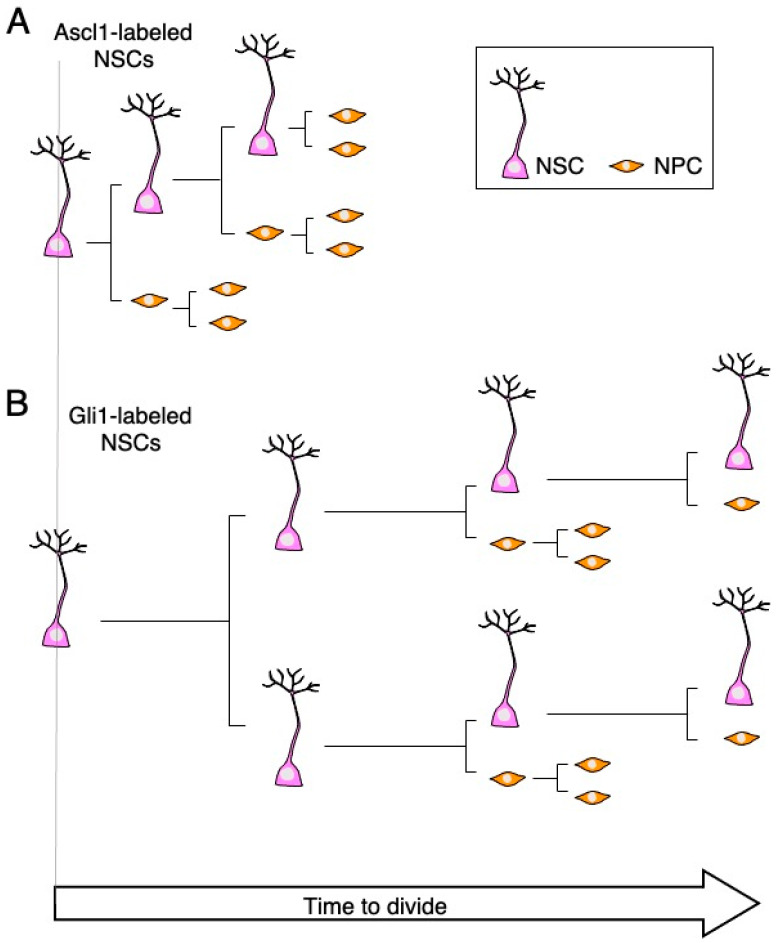
NSCs are functionally heterogeneous in the adult DG. (**A**) Ascl1-labeled NSCs undergo several asymmetric divisions before all cells differentiate into NPCs for the subsequent production of new neurons. (**B**) Gli1-labeled NSCs can undergo symmetric division first and then repeat asymmetric division with a slower cycle than Ascl1-labeled NSCs. This population conceivably contributes to the preservation of the NSC pool in the adult DG.

As described above, on the basis of phenotypic analysis using fixed brain tissue of transgenic mice, many studies have revealed that extracellular and intracellular factors are involved in the regulation of NSC behavior. Advances in two-photon microscopy have greatly propelled the studies of neural circuits and brain functions in the past decade by enabling high-resolution morphological and functional intravital imaging of the brain. This advance also enables chronic in vivo imaging to track the fate of individual NSCs over time in the adult DG. A recent study showed that Ascl1-expressing NSCs mostly divide asymmetrically and generate an NSC and a cell committed toward the neuronal lineage [56]. This population repeats the asymmetric division a few times within about 10 days, and then eventually, all of the cells turn into neurons [38,56,57] (Figure 2A). Subsequent studies discovered another population of NSCs, which are expressing Gli1 [38,43,58] (Figure 2B). They have properties distinct from those of the Ascl1-expressing NSC population, i.e., Gli1-expressing NSCs can keep the potential for long-term self-renewal and asymmetrically divide at most four times in 102 days. These studies revealed the functional heterogeneity of NCSs in the adult DG, which may explain how preservation of the NSC pool and neurogenesis are balanced to ensure proper brain functions, although the underlying molecular mechanism remains elusive.

## 3. Neurogenesis from Non-Stem Cells and Future Prospects of Direct Reprogramming Technology to Treat Neurological Disorders

### 3.1. Forced Neurogenesis from Non-Stem Cells

Neuronal regeneration in the adult brain is critical for attaining functional recovery in patients afflicted with neurological diseases, including brain injury. NSCs, located in limited regions such as the SGZ and V-SVZ in the adult brain, can generate new neurons to restore lost neuronal circuits in pathological conditions. A recent study showed that astrocytes in the adult mice striatum could also behave as NSC-like cells that generate new neurons after ischemic injury [59]. However, the number of neurons newly generated from NSCs and local astrocytes is much too small for the full recovery of neuronal functions. Moreover, in neurodegenerative diseases such as Alzheimer’s disease, neuronal loss is observed in all brain regions, including non-neurogenic areas, making it even more difficult to replenish lost neurons by counting solely on the intrinsic neurogenic potential of these endogenous cells.

Transplantation of exogenous NSCs derived from human induced pluripotent stem cells has been explored as a potential therapy for neurological diseases. In animal models, transplanted NSCs can survive, proliferate, and regenerate new neurons in infarct areas, although the risks of immune rejection and tumor development still remain substantial drawbacks of this therapeutic approach. An innovative approach toward replacing damaged neurons is to directly induce fate conversion of non-neuronal cells residing in the injured brain into neurons by a process called direct reprogramming. Compared to exogenous cell transplantation, direct reprogramming has several advantages, such as a short induction period, efficient conversion, reduced tumorigenesis risk, and lack of the need for ex vivo culture.

Knowledge of extrinsic and intrinsic factors that regulate cell fate acquisition during neurogenesis was critical for the development of the field of cell reprogramming. It has become widely accepted that lineage-specific transcription factors can convert given cells into those in different lineages [60], as first exemplified by the milestone study in 1987 showing that overexpression of myogenic transcription factor MyoD induced conversion of mouse embryonic fibroblasts (MEFs) to myoblasts [61]. In 2010, direct reprogramming of MEFs into induced neuronal (iN) cells in vitro was reported; simultaneous expression of Ascl1, Brn2, and Myt1l in MEFs efficiently converted them to iN cells [11]. Most iN cells in that study exhibited properties of glutamatergic, excitatory neuronal subtype. Afterwards, many others used different combinations of transcription factors to convert MEFs into distinct neuronal subtypes, such as dopaminergic, motor, retinal, and peripheral sensory neurons [60]. Furthermore, it has also been reported that knockdown of a single gene, *polypyrimidine tract binding protein 1* (*Ptbp1*) encoding an RNA binding protein, was sufficient to induce iN cells from somatic cells in vitro [62]. These studies have indicated that the generation of iN cells from somatic cells is more feasible than previously thought and holds promise for future clinical applications.

### 3.2. Neurogenesis from Non-Neurogenic Brain-Resident Cells

Astrocytes, a glial cell type in the brain, become reactive after brain damage and eventually contribute to glial scar formation [63]. Astrocytes are considered to be one of the ideal sources for in vivo neuronal conversion because there is less concern about depletion of the starting cells after conversion due to their abundance in the brain. Direct neuronal reprogramming from astrocytes in vitro was achieved by forced expression of *Pax6*, which had been known to maintain NSC properties and to regulate neurogenesis in the embryonic forebrain [64]. Moreover, Neurog2 efficiently converts astrocytes into glutamatergic iN cells [65,66], while distal-less homeobox 2 (Dlx2) induces GABAergic neuronal conversion from astrocytes, in agreement with the distinct roles of these two factors in neuronal subtype determination during neurogenesis of NSCs [66]. In addition to Dlx2, Ascl1 is also known to induce GABAergic neuronal conversion from astrocytes and to characterize the difference between the transcriptional transitions induced by Neurog2 and Ascl1 during neuronal conversion, transcriptome analysis was conducted using *Ascl1*- and *Neurog2*-transduced astrocytes [67]. The transcriptomic changes induced by Ascl1 and Neurog2 were largely different during the time course of neuronal reprogramming, indicating that Ascl1 and Neurog2 regulate distinct neurogenic gene expression networks in the same cellular background, although these two transcription factors share some target genes, such as *Neurod4*, that are sufficient to induce functional neurons from astrocytes [67]. Taken together, these findings indicate that each reprogramming factor drives the expression of its own subset of genes to define neuronal subtypes, while both factors induce in common some important genes for neuronal conversion from astrocytes.

Microglia, the major immune cells in the adult brain, are derived from primitive macrophages [68], which arise from early erythro–myeloid progenitors in the yolk sac during the early embryonic stage [69,70,71]. With the establishment of the blood circulation, these primitive macrophages migrate into the developing CNS, where the combination of ontogeny and the CNS environment confers the microglial signature on the migrated cells [72,73,74]. Microglia converge at injured sites and become a predominant cell type within the glial scar [75,76]. Furthermore, microglia self-renew and rapidly repopulate when almost depleted in the adult mouse brain [77]. Therefore, microglia are considered to be another ideal source to be converted into neuons without exhaustion in the lesion site.

**Figure 3:**
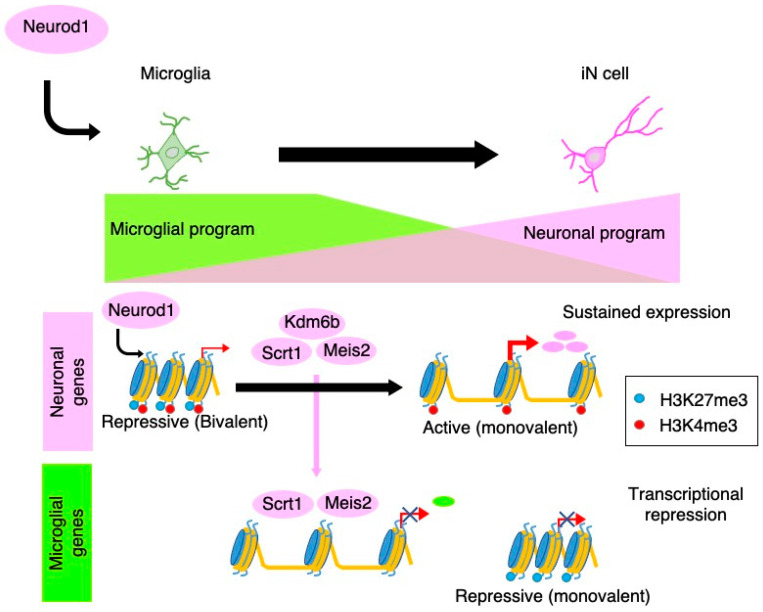
Molecular mechanisms underlying Neurod1-mediated neuronal conversion from microglia. NeuroD1 associates with closed chromatin regions with bivalent histone modifications (H3K4me3 and H3K27me3) in microglia to induce neuronal gene expression. These regions are resolved to a monovalent H3K4me3 mark at later stages of reprogramming to establish the neuronal identity. NeuroD1 also suppresses microglial gene expression through the induction of transcriptional repressors (Scrt1 and Meis2). In parallel, the microglial epigenetic signature in promoter and enhancer regions is erased, resulting in the elimination of microglial identity.

We have recently demonstrated that microglia can be directly converted into neurons by the expression of a single transcription factor, *neurogenic differentiation 1* (*NeuroD1*) [78] (Figure 3).

Transcriptomic analysis during the reprogramming showed that the gene expression pattern of iN cells converted from microglia strongly resembled that of actual neurons. We also found that NeuroD1 accesses and induces the expression of neuronal genes harboring closed chromatin configuration with bivalent histone modifications, i.e., active (trimethylation of histone H3 at lysine 4 (H3K4me3)) and repressive (H3K27me3) marks, in microglia. After NeuroD1 binding, these bivalent chromatin regions are resolved to a monovalent active state (H3K4me3), at least in part, through the induction of *lysine (K)-specific demethylase 6B* (*Kdm6b*), which induces demethylation of H3K27. NeuroD1 also induces transcriptional repressors, scratch family zinc finger 1 (Scrt1), and Meis homeobox 2 (Meis2) to suppress the expression of transcription factors critical for microglia-specific gene expression, leading to the elimination of microglial identity. Thus, microglia lose the microglial identity and establish the neuronal identity after transduction with NeuroD1.

As we described above, distinct reprogramming-inducing factors affect different neuronal gene expressions and neuronal subtypes. The characteristic differences of the response to conversion-inducing factors depending on the original cell source must also be taken into account to effectively obtain desired neuronal subtypes. For instance, Ascl1 preferentially occupies regions associated with trivalent histone modifications (H3K4me1, H3K27ac, and H3K9me3) in MEFs to induce the expression of neuronal genes [79]. Ascl1 fails to induce neuronal reprogramming from keratinocytes due to the absence of such a trivalent state on Ascl1 target sites in the cells. Furthermore, NeuroD1 is able to convert oligodendrocytes but not non-reactive astrocytes into iN cells in vitro [78]. This is because oligodendrocytes but not non-reactive astrocytes have a bivalent chromatin signature (H3K4me3 and H3K27me3) in NeuroD1-targeted loci around neuronal genes. In the case of Neurog2-mediated neuronal reprogramming from astrocytes, prolonged culture increased H4K20me3 levels in Neurog2 target genes, modifying the local chromatin environment so that it becomes favorable for binding of the transcription repressive complex REST. Consequently, Neurog2 became unable to access the NeuroD4 promoter and could not induce neuronal reprogramming. These findings indicate that cell-type-specific epigenetic profiles, such as histone modifications, control the accessibility of each neuronal reprogramming factor to target genes and affect reprogramming efficiency.

### 3.3. Therapeutic Potential of Intravital Neuronal Reprogramming from Brain-Resident Non-Neuronal Cells

**Figure 4:**
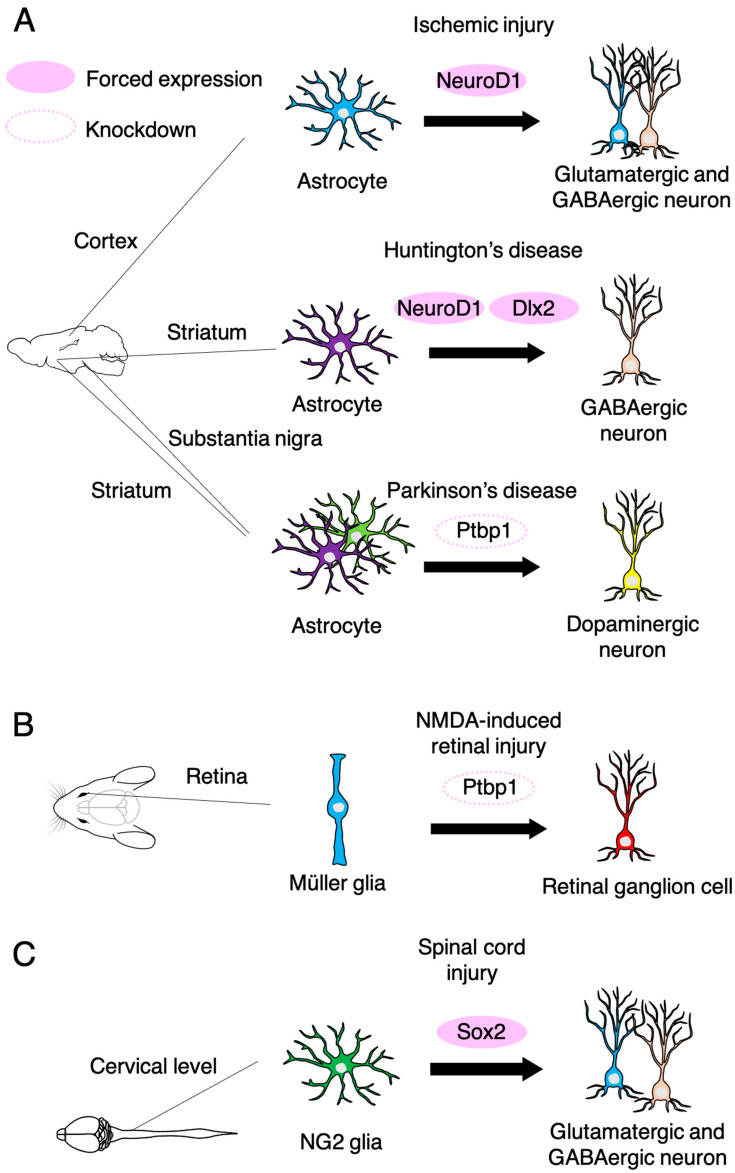
In vivo neuronal reprogramming from non-neuronal cells in the adult brain, retina and spinal cord. (**A**) NeuroD1 converts reactive astrocytes to both glutamatergic and GABAergic neurons in the cortex after ischemic stroke induced by cortical injection of endothelin-1, leading to neurological recovery. In Huntington’s disease model mice, combinatorial expression of NeuroD1 and Dlx2 converts striatal astrocytes into GABAergic neurons and restores motor function. Downregulation of Ptbp1 converts striatal astrocytes into dopaminergic neurons, inducing motor functional recovery. (**B**) Ptbp1 downregulation converts Müller glia into retinal ganglion cells in NMDA-induced retinal injury model mice, and thereby, repairs the visual function. (**C**) Forced expression of Sox2 converts NG2 glia into both glutamatergic and GABAergic neurons in the injured spinal cord, promoting functional recovery.

Recent studies have achieved in vivo direct neuronal reprogramming from endogenous astrocytes and oligodendrocyte progenitor cells within several mouse brain regions and the spinal cord. We have also reported that NeuroD1 can convert microglia to DARPP32-positive striatal projection neuron (SPN)-like cells in the adult mouse striatum, and these iN cells were functionally integrated into the brain circuits through synaptic connections with other neurons [78]. More recent research has shown the conversion of astrocytes to iN cells in a focal ischemia model induced by the vasoconstrictive peptide endothelin-1, resulting in 30–40% regeneration of lost neurons in the motor cortex of adult mice and in the improvement of neurological dysfunctions [80] (Figure 4A). In Huntington’s disease model mice, combinatorial expression of NeuroD1 and Dlx2 in striatal astrocytes induces conversion into GABAergic neurons. These iN cells exhibit action potential, synaptic events, and axonal projection to the globus pallidus and substantia nigra, as normal SPNs do, leading to increased longevity and improved motor functions [81]. In vivo *Ptbp1* knockdown has recently been reported to directly convert astrocytes in the striatum or substantia nigra into dopaminergic neurons, which reverses motor neuron dysfunction in chemically induced Parkinson’s disease model mice [82,83]. Moreover, *Ptbp1* downregulation converts Muller glia into functional retinal ganglion cells in the adult retina with high efficiency. Converted retinal ganglion cells project to the dorsal lateral geniculate nucleus and superior colliculus and restore visual impairment [83] (Figure 4B). NG2 glia are neural cells that are distinct from neurons, astrocytes, oligodendrocytes, and microglia, and are identified by the expression of proteoglycan NG2 [84]. Qian et al. reported that NG2 glia exhibits neurogenic potential in the injured but not the intact spinal cord. Although endogenous Sox2 is required for spinal cord injury (SCI)-induced transient reprogramming of NG2 glia to neurons, ectopic Sox2 expression is sufficient to confer the full neurogenic potential on NG2 glia [85] (Figure 4C). The generated neurons are inhibitory or excitatory neurons and connect with the local network of propriospinal neurons, promoting functional recovery after SCI [85]. These results suggest that direct in vivo reprogramming will be a practical application for addressing unmet medical needs such as treatments for ischemic injury, SCI, Huntington’s disease, Parkinson’s disease, and retinal degenerative disease. Further investigations, e.g., investigations using non-human primate models, will be needed to achieve clinical translation.

## 4. Conclusions

Despite substantial advances in understanding numerous signaling pathways, little is known about how NSCs integrate many diverse signals to ultimately make choices between retaining quiescence vs. entering activation, and dividing symmetrically vs. asymmetrically. Moreover, it is far from clear why NSCs cannot preserve their pool as age advances. Future research will need to focus on interactions between signaling pathways in order to identify the hubs and on the hierarchy that coordinates incoming signals. Nevertheless, strategies to boost neurogenesis from NSCs and induction of neurogenesis from brain resident non-neuronal cells by direct reprogramming hold great promise as potential therapeutic strategies. Further optimization of reprogramming factors and cell sources appropriate for each CNS disease pathology will bring direct reprogramming technology one step closer to clinical application.

## Figures and Tables

**Table 1:** List of extrinsic factors regulating NSC quiescence and activation.

Extrinsic Factor	Effect on NSC State	Source in the Niche	Reference
Jag1	Quiescence	NPCs and granule neurons	[13]
Notch	Quiescence	NSCs	[14,15]
Wnt	Activation	NSCs and Astrocytes	[17,18]
BMP	Quiescence	Granule neurons and NSCs	[19,20]
Mfge8	Quiescence	NSCs and astrocytes	[21]
Shh	Activation	Mossy fibers	[24]
GABA	Quiescence	PV interneurons	[26,27,28]
DBI	Quiescence	NSCs	[26,29]
Glutamate	Activation	Astrocytes	[26,30]
Itgb1	Quiescence	NSCs	[31,32]

**Table 2:** List of intrinsic factors regulating NSC quiescence and activation.

Intrinsic Factor	Effect on NSC State	Function	Reference
Ascl1	Activation	Upregulation of genes associated with cell cycle progression	[35]
Hes	Quiescence	Transcriptional repression of *Ascl1*	[36,37]
Id	Quiescence	Destabilization of Ascl1 by sequestering its dimerization partner E47	[15,39]
Huwe1	Quiescence	Proteasomal degradation of Ascl1	[40]
Ilk	Quiescence	Inhibition of Akt/mTOR signaling	[31,32]
Pten	Quiescence	Suppression of PI3K/Akt/mTOR pathway	[41,42,43,50]
PI3K/Akt/mTOR	Activation	Inactivation of Foxo3 and upregulation of genes associated with cell cycle progression	[21,41,42,44,46,50]
Foxo3	Quiescence	Transcriptional repression of Ascl1 target genes	[48]

## Data Availability

Not applicable.
